# Communicating the diagnosis of a hematological neoplastic disease to patients’ minor children: a multicenter prospective study

**DOI:** 10.1093/oncolo/oyae104

**Published:** 2024-05-22

**Authors:** Beatrice Manghisi, Lorenza Borin, Maria Rosaria Monaco, Gaia Giulia Angela Sacco, Laura Antolini, Raffaele Mantegazza, Monica Barichello, Umberto Mazza, Patrizia Zappasodi, Francesco Onida, Luca Arcaini, Roberto Cairoli, Carlo Gambacorti Passerini

**Affiliations:** Hematology Division, Fondazione IRCCS San Gerardo dei Tintori, Monza, Italy; Department of Medicine and Surgery, University of Milano-Bicocca, Milan, Italy; Hematology Division, Fondazione IRCCS San Gerardo dei Tintori, Monza, Italy; Hematology Division, Fondazione IRCCS San Gerardo dei Tintori, Monza, Italy; Department of Medicine and Surgery, University of Milano-Bicocca, Milan, Italy; Department of Medicine and Surgery, University of Milano-Bicocca, Milan, Italy; Department of Medicine and Surgery, University of Milano-Bicocca, Milan, Italy; Department of Mental Health, Clinical Psychology, ASST Grande Ospedale Metropolitano Niguarda, Milan, Italy; Department of Mental Health, Clinical Psychology, ASST Grande Ospedale Metropolitano Niguarda, Milan, Italy; Division of Hematology, Fondazione IRCCS Policlinico San Matteo, Pavia, Italy; Hematology Division, Policlinico di Milano Ospedale Maggiore – Fondazione IRCCS Ca Granda, Milan, Italy; Division of Hematology, Fondazione IRCCS Policlinico San Matteo, Pavia, Italy; Department of Medicine and Surgery, University of Milano-Bicocca, Milan, Italy; Division of Hematology, Oncology and Molecular Medicine, Niguarda Cancer Center, ASST Grande Ospedale Metropolitano Niguarda, Milan, Italy; Hematology Division, Fondazione IRCCS San Gerardo dei Tintori, Monza, Italy; Department of Medicine and Surgery, University of Milano-Bicocca, Milan, Italy

**Keywords:** diagnosis, disease communication, hematological malignancy, family well-being, doctor-patient relationship

## Abstract

**Background:**

When a hematological malignancy is diagnosed, the whole family carries the burden of the disease; parents often try to protect minor children from suffering by avoiding communication about their disease. Since 2009, patients with minors at the Adult Hematology Division at San Gerardo Hospital (Monza) can take part in the “Emanuela Project”: children can visit parents and talk with psychologists and hematologists, who explain the disease through simple metaphors.

**Materials and Methods:**

The EMY STUDY aimed to evaluate the impact of illness-related communication on children’s behavior, comparing Monza’s experience with other Hematology Units, where the communication is delegated to parents or psychological support. Questionnaires exploring the children’s main behaviors (school performance, appetite, sleeping patterns, attachment to family figures, and family dialogue) were administered to both sick (SP) and healthy (HP) parents. From 2017 to 2021, 32 patients were enrolled, 20 from Monza and 12 from other hospitals; 84 questionnaires were globally collected.

**Results:**

In Monza’s group, no major changes in children’s behavior were observed and an open dialogue about the disease was often possible. Disease communication is considered crucial and perceived as a responsibility of parents together with a professional figure, mainly the hematologist. Patients were satisfied with “Emanuela Project,” reporting positive effects on doctor-patient relationship. Difficulties in separation were significantly higher at other hospitals (*P* = .019) than in Monza. While at other centers communication is considered parents’ responsibility, Monza’s patients emphasize the role of professional figures (*P* = .007). Differently from other hospitals, the role of the hematologist is crucial to Monza’s patients (*P* = .001).

**Conclusion:**

Disease communication to patients’ offspring is a crucial moment in the process of care, and the hematologist can play a major role in this difficult task, with potential positive effects both on children’s well-being and on doctor-patient relationship.

## Teaser

The EMY STUDY evaluated the impact of illness-related communication on children’s behavior, comparing the Monza experience with other hematology units where the communication is delegated to parents or psychological support.

**Implications for Practice**

This first-in-its-kind study provides impactful and practical information about the importance of diagnosis communication to the whole patient’s family. Minor children should be informed about a parent’s hematological disease with proper communication skills, based on the use of simple images and metaphors. Physicians are currently asked to reconsider their role in the process of care: hematologists should not delegate this difficult task to parents or psychological support, as they can play a crucial role in communication with patients’ offspring, with a potential positive impact on children’s well-being, family dialogue, and doctor-patient relationship.

## Introduction

The diagnosis of a hematological neoplastic disease bears a great impact both on the patient and on the functioning of the family unit. The entire family carries the burden of the disease, often experiencing major abrupt changes in lifestyle and relationships.^1-4^

The onset of acute leukemia represents a life-threatening condition, requiring immediate hospitalization and urgent medical care; aggressive lymphomas and myelomas with high disease burden can also have a severe clinical presentation, needing intensive treatment. In both cases, hospitalization periods can be long, due to close treatment schedules, side effects of chemotherapies, and infectious complications.^5^

In Medical Oncology, the impact of a parent’s disease on the family has been widely studied,^6-9^ while the peculiarity of Hematological malignancies was never clearly explored.^10,11^

At the conclusion of the diagnostic process, the clinician informs the patient about the diagnosis and prognosis, explaining therapeutic options and answering questions. This is a crucial moment in the development of a therapeutic relationship, and an open dialogue is needed not only about clinical aspects, but also about personal and familial issues.^12^

Children and adolescents living with an affected parent often represent the “forgotten voice” within the family.^1,13-15^ Parents tend to avoid communication about the disease with their children to protect them from suffering, believing that they would not understand.^13,16,17^ Conversely, the most recent literature underlines the importance of involving children in this process and encourages an open dialogue about the disease.^9,18-22^

Hematologists frequently delegate this communication with children to psychologists.^4,16,20,23^ In the present study, we examined the issue of communicating information about the disease to patients’ offspring and investigated the role of the hematologist in this task.^1,24^

Since 2009, at the Adult Hematology Unit of San Gerardo Hospital (Monza, Italy), patients with minors can take part in the “Emanuela Project,” an intervention which allows children to visit their hospitalized parent, and talk with a hematologist and a psychologist. The physician explains the disease using simple images, while the psychologist helps to express emotions and fears.

Throughout the years, we received overall positive feedback from patients about the “Emanuela Project”; however, we recently felt the need to further explore the impact of our intervention on both children and the whole family, to understand the importance of the hematologist in communication and to compare our experience with other Hematology Units in Northern Italy.

## Aims

The primary objective of the study was to evaluate the impact of illness-related communication on children’s health status and behavior, comparing our experience in “Emanuela Project” with other Hematology Units where the communication is delegated to parents or psychological support.

The secondary objectives of the study were

(1) to evaluate the impact of illness-related communication on doctor-patient relationship and patient’s compliance to therapy and
(2) to explore the role of the hematologist in illness-related communication with children.

## Methods

In 2017, we designed “EMY STUDY,” a prospective observational multicenter study, recruiting patients aged >18 years, with a new diagnosis (31 patients) or relapse (1 patient) of hematological neoplastic disease and at least 1 minor child (0-18 years) at the time of enrollment.

Four Hematology Units in Northern Italy took part in the study, after approval by the Ethical Committee: San Gerardo Hospital in Monza, Niguarda Hospital in Milano, Policlinico Hospital in Milano, and Policlinico San Matteo Hospital in Pavia.

All 4 centers involved in the study have a wealth of experience in the management of acute hematological conditions and treat a significant number of patients each year: the median number of new acute leukemia diagnoses is 60 per year for San Gerardo Hospital in Monza, 60 per year for Niguarda Hospital in Milan, 35 per year for Policlinico Hospital in Milan, and 55 per year for Policlinico San Matteo Hospital in Pavia.

### Communication methods

We involved in the study some Hematology Units with different approaches to illness-related communication with patients’ offspring, in order to compare Monza’s experience with other settings. We hereby provide a brief description of different communication methods:

(1) At San Gerardo Hospital (Monza), the “Emanuela Project” allows children and adolescents to visit their ill parent and to talk with a hematologist and a psychologist. It usually takes place in a dedicated room close to the Hematology Ward, during the first week of hospitalization, after the diagnosis has been explained to the patient. The whole family, a hematologist, and a psychologist are involved in the interview. The physician explains the disease and the therapies using simple images and metaphors. The metaphor of the “flower garden,” derived from pediatric experience at San Gerardo Hospital (Dr. Jankovic),^1,25^ is used to explain acute leukemia (Supplementary Figure S1): the bone marrow is described as a flowery meadow and leukemia blasts are represented as weeds growing in the meadow; the hematologist uses a special herbicide (chemotherapy), which leaves the meadow empty during a phase called aplasia, allowing the growth of new healthy flowers. On the back of this image, we created new metaphors for other clinical situations: the image of a factory with broken gears is used to explain myelodysplastic syndromes (Supplementary Figure S2), the image of a greenhouse, protecting flowers from bad weather, helps to understand the necessity of hospitalization in a clean room (Supplementary Figure S3); to explain the choice of donor in bone marrow transplantation and post-transplant complications such as graft-versus-host disease, we use the metaphor of soccer teams wearing different uniforms, representing the donor’s immune system fighting against recipient’s tissues. The psychologist helps children to express emotions and fears by open questions, playing, or painting, according to their age. At the end of the intervention, children can visit the Hematology Unit, including their parent’s room.
(2) At Niguarda Hospital (Milano), patients with minors are offered the opportunity of a disease communication to their children; although both physicians and psychologists can offer this possibility, the major role in the communication is played by psychologists. The communication usually takes place in a doctor’s office, separated from the Hematology Unit. Specific images and communication methods are not used.
(3) At Policlinico Hospital (Milano) and Policlinico San Matteo Hospital (Pavia), there is no established communication with patients’ offspring; patients are offered psychological support during the treatment course, but the task of disease communication to minor children is left to parents.

### Data collection and analysis

In 2017, at the time of study design, a team of hematologists, psychologists, and statisticians developed a questionnaire consisting of multiple-choice questions and brief open questions, exploring changes in children’s behavior and parents’ opinion about disease communication. In the process of questionnaire development, a thorough literature review was initially conducted, aiming to analyze existing tools commonly used in this kind of research. Hematologists and psychologists with great experience in communicating diagnosis with children defined the main areas of interest in children’s behavior, considering the expected impact of the intervention, and developed the structure of the questionnaire; statisticians helped in organizing clear and well-defined multiple-choice questions.

Questionnaires consisted of 3 parts:

(1) Part 1, filled in by study investigators, included demographic and clinical data (patient’s and children’s age, diagnosis, and duration of hospital stay).
(2) Part 2, filled in by both parents, explored changes in children’s behavior, dialogue about the disease, and parents’ opinion about disease communication with minors.
(3) Part 3, filled in by both parents, was dedicated only to San Gerardo Hospital patients, as it specifically explored opinion about Emanuela Project, figures involved, communication methods, and the impact on the family unit.

Questionnaires were administered 30-60 days after the communication of diagnosis. The choice of a certain time point depended on the expected mean duration of hospital stay for patients with a new diagnosis of hematological malignancy, in order to allow both parents to get an accurate view of modifications in their children’s behavior. Each parent was asked to complete a different questionnaire per child in case of multiple children.

Data were analyzed by the statistical software STATA; answers given by sick and healthy parent were analyzed separately, as well as answers given by Monza’s patients and other hospitals’ ones. At the time of study design, the sample size was determined using an apriori power analysis.

Mc Nemar statistical test was used to compare answers given by healthy (HP) and sick parents (SP) in the Monza’s group; chi-square statistical test was used to compare results collected from Monza and other hospitals’ patients. A “*P*-value” <.05 was considered statistically significant.

## Results

### Sample description: epidemiological and clinical information

Between November 2017 and March 2021, 32 patients were enrolled, 17 females (53.1%) and 15 males (46.9%); 20 patients were recruited from San Gerardo Hospital in Monza, 7 from Niguarda Hospital in Milano, 4 from Policlinico Hospital in Milano, and 1 from San Matteo Hospital in Pavia.

Mean patients’ age at diagnosis was 45.1 years (SD 7.7, range 30-66); most patients were Italian (91%), with a little representation of other nationalities in the sample (3% Bulgarian, 3% Romanian, and 3% Venezuelan).

Acute leukemia represented the most frequent diagnosis, accounting for 59.4% of cases (46.9% myeloid and 12.5% lymphoblastic), followed by non-Hodgkin lymphoma (21.9%), multiple myeloma (9.4%), aplastic anemia (6.3%), and chronic myeloid leukemia (3.1%). (Supplementary Figure S4)

Mean duration of hospitalization was 30.3 days, with a wide range of distribution (SD 18.8, range 5-120) due to the heterogeneity of diseases.

Children involved were 51, with an overall mean age of 9.5 (SD 4.9, range 1-18). Mean age was 11.2 years (SD 4.9, range 4-18) for the eldest child, 7.1 years (SD 3.4, range 1-14) for the second born child; there was only a third-born child, aged 2. All the children were Italian speaking.

Each parent (both the ill and the healthy ones) was asked to complete a questionnaire per child; 84 questionnaires were globally collected, with some data from healthy parents missing because of difficulty in data collection, especially during the COVID-19 pandemic. Missing healthy parent questionnaires were 1/20 at Monza and 6/12 at other hospitals.

Table 1 summarizes the main characteristics of patients and children involved, describing the whole population and the subgroups of Monza’s and other hospitals’ patients.

**Table 1.** Patients’ characteristics.

Measure	All patients, *n* = 32 (100 %)	Monza patients, *n* = 20 (62.5%)	Other hospitals’ patients, *n* = 12 (37.5%)
Patients mean age at diagnosis (years)	45.13 (SD 7.69)	46.55 (SD 8.67)	42.75 (SD 5.17)
Patients gender			
Male	15 (46.88%)	10 (50%)	5 (41.67%)
Female	17 (53.15%)	10 (50%)	7 (58.33%)
Patients nationality			
Italian	29 (90.63%)	19 (95%)	10 (83.33%)
Bulgarian	1 (3.13%)	0 (0%)	1 (8.33%)
Romanian	1 (3.13%)	1 (5%)	0 (0%)
Venezuelan	1 (3.13%)	0 (0%)	1 (8.33%)
Children mean age (years)			
1 child	11.15 (SD 4.87)	12.21 (SD 4.3)	9.92 (SD 5.38)
2 child	7.13 (SD 3.38)	7.1 (SD 2.85)	7.2 (SD 4.66)
3 child	2 (SD 0)	2 (SD 0)	—
Hospital			
Monza	20 (62.50%)	20 (100%)	0 (0%)
Other hospitals	12 (37.50%)	0 (0%)	12 (100%)
Duration of hospitalization (days)	30.26 (SD 18.8)	31.16 (SD 23.7)	28.83 (SD 6.41)
Diagnosis			
SAA	2 (6.25%)	2 (10%)	0 (0%)
ALL	4 (12.5%)	1 (5%)	3 (25%)
AML	15 (46.88%)	10 (50%)	5 (41.67%)
CML	1 (3.13%)	0 (0%)	1 (8.33%)
NHL	7 (21.88%)	5 (25%)	2 (16.67%)
MM	3 (9.38%)	2 (10%)	1 (8.33)

Abbreviations: SAA, severe aplastic anemia; ALL, acute lymphoblastic leukemia; AML, acute myeloid leukemia; CML, chronic myeloid leukemia; NHL, non-Hodgkin lymphoma; MM, multiple myeloma.

Children were informed about the nature of the diagnosis in 98% of cases, 100% at Monza and 93.9% at other hospitals. For 82.1% of children in the whole population, it was possible to visit their ill parent during hospitalization (91% for Monza’s patients and 50% for other hospitals’ patients).

Mean frequency of visits was 1.4 times per week (SD 1.3, range 0.5-7). Most visits took place in a dedicated room (74%); less frequently, children visited parents in their hospital room (22%), and in rare cases visits were allowed only through sterile room’s window (4%). (Supplementary Figure S5)

### Impact of diagnosis communication according to both parents: Monza’s patients analysis

To evaluate the impact of our communication strategy according to both parents, we analyzed Monza patients’ data, considering answers given by SP and by the HP separately. The decision of restricting this analysis to Monza’s patients was due to the lack of many healthy parents’ data from other centers, making it difficult to perform a proper and reliable comparison.

Data from multiple-choice questions exploring changes in children’s behavior suggest that, according to both parents, there was no relevant worsening in school performance, appetite, and sleeping patterns, after communication of parent’s disease. No relevant increase of nightmares, solitary attitudes, or questions about death was reported.

Interestingly, a relevant portion of parents reported an increase in children’s attachment to family figures, both to sick parent (50% in SP opinion and 56.3% in HP opinion) and to the healthy one (41.2% in SP opinion and 53.1% in HP opinion). Moreover, 32.4% of SP and 40.6% of HP reported a difficulty in children’s separation from main family figures (Table 2).

**Table 2.** Monza patients’ analysis—children behavior.

	Sick parent’s opinion, *n* (%)	Healthy parent’s opinion, *n* (%)	Mc Nemar test (*P*-value)
Children school performance			
Unchanged	22 (73.33%)	22 (73.33%)	.625
Improved	5 (16.67%)	2 (6.67%)	
Worsened	3 (10%)	6 (20%)	
Children sleep patterns			
Unchanged	30 (88.24%)	27 (84.38%)	.375
Improved	0 (0%)	0 (0%)	
Worsened	4 (11.76%)	5 (15.63%)	
Children appetite			
Unchanged	25 (75.76%)	24 (75%)	.625
Increased	4 (12.12%)	2 (6.25%)	
Reduced	4 (12.12%)	6 (18.75%)	
Attachment to sick parent			
Unchanged	17 (50%)	14 (43.75%)	.375
Increased	17 (50%)	18 (56.25%)	
Attachment to healthy parent			
Unchanged	20 (58.82%)	15 (46.88%)	.180
Increased	14 (41.18%)	17 (53.13%)	
Difficulties in separation from family members			
Unchanged	23 (67.65%)	19 (59.38%)	.219
Increased	11 (32.35%)	13 (40.63%)	
Increased play alone			
No	26 (83.87%)	25 (89.29%)	1.00
Yes	5 (16.13%)	3 (10.71%)	
Increased nightmares			
No	27 (87.1%)	24 (77.42%)	.453
Yes	4 (12.9%)	7 (22.58%)	
Increased questions about death			
No	29 (87.88%)	24 (77.42%)	.375
Yes	4 (12.12%)	7 (22.58%)	

Table 3 shows data concerning the possibility of a dialogue about the parent’s disease in the family unit. An open dialogue about the disease was possible “always” or “often” according to the majority (82.4% of SP and 84.4% of HP); 100% of parents reported that it was never necessary to hide hospitalization or outpatient visits to their offspring; most parents stated that it was never or rarely necessary to hide side effects of therapies (91% of SP and 84.4% of HP). 52.9% of SP and 43.8% of HP believe that children were worried about sick parent’s life; 25% of SP and 26.7% of HP think that children were also worried about the life of healthy parents.

**Table 3.** Monza patients’ analysis—family dialogue about the disease.

	Sick parent’s opinion, *n* (%)	Healthy parent’s opinion, *n* (%)	Mc Nemar test (*P*-value)
Children want information about course of disease			
Never	4 (11.76%)	2 (6.25%)	.109
Sometimes	18 (52.94%)	14 (43.75%)	
Often	8 (23.53%)	6 (18.75%)	
Always	4 (11.76%)	10 (31.25%)	
Children want to talk about the disease			
Never	10 (29.41%)	9 (28.13%)	1.00
Sometimes	19 (55.88%)	19 (59.38%)	
Often	3 (8.82%)	1 (3.13%)	
Always	2 (5.88%)	3 (9.38%)	
Children talk about disease outside family			
Never	7 (21.88%)	11 (34.38%)	1.00
Sometimes	20 (62.5%)	18 (56.25%)	
Often	4 (12.5%)	1 (3.13%)	
Always	1 (3.13%)	2 (6.25%)	
Children talk about disease with family members			
Never	8 (25%)	10 (31.25%)	.625
Sometimes	15 (46.88%)	17 (53.13%)	
Often	9 (28.13%)	4 (12.5%)	
Always	0 (0%)	1 (3.13%)	
Free dialogue about the disease in the family			
Never	0 (0%)	0 (0%)	1.00
Sometimes	6 (17.65%)	5 (15.63%)	
Often	7 (20.59%)	5 (15.63%)	
Always	21 (61.76%)	22 (68.75%)	
Need to hide parent’s visits and hospitalization			
Never	34 (100%)	32 (100%)	1.00
Sometimes	0 (0%)	0 (0%)	
Often	0 (0%)	0 (0%)	
Always	0 (0%)	0 (0%)	
Need to hide side effects of therapies			
Never	23 (67.65%)	20 (62.5%)	.625
Sometimes	8 (23.35%)	7 (21.88%)	
Often	2 (5.88%)	5 (15.63%)	
Always	1 (2.94%)	0 (0%)	
Children fear for sick parent’s life			
No	16 (47.06%)	18 (56.25%)	.344
Yes	18 (52.94%)	14 (43.75%)	
Children fear for healthy parent’s life			
No	24 (75%)	22 (73.33%)	.688
Yes	8 (25%)	8 (26.67%)	

Some questions explored parents’ opinion about the opportunity of explaining the disease to children and about the involvement of professional figures in communication (Supplementary Table S1). Almost all parents agree that it is important to share information with their children about the disease (94.1% of SP and 100% of HP). Communication is often perceived as a responsibility of parents together with a specific professional figure (59.4% of SP, 67.7% of HP). Interestingly, most parents believe that the hematologist, who takes care of the patient, can play a relevant role in communication with children (94.1% of SP and 96.9% of HP).

Part 3 of the questionnaire specifically explores opinions about the Emanuela Project (Table 4). Almost all parents were satisfied with the intervention; an overall very positive opinion was collected about the communication methods and the role of the hematologist and the psychologist. Interestingly, 81.3% of SP and 83.9% of HP reported that simple metaphors used with children improved their own comprehension of the disease; moreover, 79.4% of SP and 82.1% of HP believe that the Emanuela Project improved their relationship with the medical staff, with potential positive implications on compliance to therapies.

**Table 4.** Monza patients’ analysis—parents’ opinion about Emanuela Project.

	Sick parent’s opinion, *n* (%)	Healthy parent’s opinion, *n* (%)	Mc Nemar test (*P*-value)
Did the hematologist explain the disease in a simple way?			
No	0 (0%)	0 (0%)	1.00
Doesn’t know	2 (5.88%)	2 (6.25%)	
Yes	32 (94.12%)	30 (93.75%)	
Opinion about communication methods used			
Negative	0 (0%)	0 (0%)	1.00
Doesn’t know	1 (3.13%)	2 (6.25%)	
Positive	31 (96.88%)	30 (93.75%)	
Was the hematologist useful in the intervention?			
No	0 (0%)	0 (0%)	1.00
Doesn’t know	2 (6.06%)	2 (6.45%)	
Yes	31 (93.94%)	29 (93.55%)	
Was the psychologist useful in the intervention?			
No	4 (12.12%)	4 (13.79%)	.031
Doesn’t know	1 (3.03%)	3 (10.34%)	
Yes	28 (84.85%)	22 (75.86%)	
Was the intervention useful overall?			
No	0 (0%)	0 (0%)	1.00
Doesn’t know	2 (5.88%)	2 (6.25%)	
Yes	32 (94.12%)	30 (93.75%)	
Considering the intervention, is it correct to communicate diagnosis to children?			
No	2 (5.88%)	0 (0%)	1.00
Doesn’t know	0 (0%)	1 (3.13%)	
Yes	32 (94.12%)	31 (96.88%)	
Improvement in parents’ comprehension of the disease thanks to images/metaphors			
No	4 (12.5%)	2 (6.45%)	.685
Doesn’t know	2 (6.25%	3 (9.68%)	
Yes	26 (81.25%)	26 (83.87%)	
Improvement in relationship with medical staff thanks to the intervention			
No	6 (17.65%)	3 (10.71%)	1.00
Doesn’t know	1 (2.94%)	2 (7.14%)	
Yes	27 (79.41%)	23 (82.14%)	

No significant differences were observed in answers given by SP and HP, suggesting that parents’ perception of children’s behavior did not differ, despite the patient’s long hospitalization periods. The only significant difference between SP and HP opinions was observed about the role of the psychologist in the Emanuela Project (*P* = .031): SP considered it more useful than HP.

### A multicenter comparison of opinions: Monza’s patients versus other hospitals’ ones

A separate statistical analysis compared answers given by Monza’s patients versus those treated at other hospitals. Only questionnaires fulfilled by SP were considered, given the lack of several healthy parents’ data from other centers.

Most items of the questionnaire exploring children’s behavior did not point out any relevant difference between the 2 groups (Table 5). Two items showed some relevant findings: a significantly higher percentage of other hospitals’ patients reported an increased attachment to healthy parent, compared to Monza’s patients (75% vs 41.2%, *P* = .027). Similarly, a greater difficulty in separation from family figures was noticed in other hospitals’ sample than in Monza’s one (66.7% vs 33.4%, *P* = .019).

**Table 5.** Monza versus other hospitals analysis—children behavior.

	Monza SP opinion, *n* (%)	Other centers SP opinion, *n* (%)	Chi-square test (*P*-value)
Children school performance			
Unchanged	22 (73.33%)	9 (75%)	.602
Improved	5 (16.67%)	1 (8.33%)	
Worsened	3 (10%)	2 (16.67%)	
Children sleep patterns			
Unchanged	30 (88.24%)	10 (83.33%)	.284
Improved	0 (0%)	0 (0%)	
Worsened	4 (11.76%)	2 (16.67%)	
Children appetite			
Unchanged	25 (75.76%)	11 (91.67%)	.675
Increased	4 (12.12%)	0 (0%)	
Reduced	4 (12.12%)	1 (8.33%)	
Attachment to sick parent			
Unchanged	17 (50%)	3 (27.27%)	.138
Increased	17 (50%)	8 (72.73%)	
Attachment to healthy parent			
Unchanged	20 (58.82%)	3 (25%)	.027
Increased	14 (41.18%)	9 (75%)	
Difficulties in separation from family members			
Unchanged	23 (67.65%)	4 (33.33%)	.019
Increased	11 (32.35%)	8 (66.67%)	
Increased play alone			
No	26 (83.87%)	12 (100%)	.125
Yes	5 (16.13%)	0 (0%)	
Increased nightmares			
No	27 (87.1%)	12 (100%)	.176
Yes	4 (12.9%)	0 (0%)	
Increased questions about death			
No	29 (87.88%)	11 (91.67%)	.675
Yes	4 (12.12%)	1 (8.33%)	

Abbreviation: SP, sick parent.

Concerning family dialogue (Supplementary Table S2), a greater proportion of Monza’s patients reported that children tended to talk about the disease with family members “often” or “always,” compared to other hospitals’ group (28% vs 0%); despite not meeting the criteria for statistical significance (*P* = .067), due to the small sample size, we consider this finding quite relevant. We also observed that children tended to be more worried about both parents’ life in the Monza’s group, compared to other centers: this finding could be a consequence of the open family dialogue about the disease in Monza’s group, where children easily express fears and concerns.

Finally, significant findings were observed about disease communication and professional figures involved (Table 6). All patients agree that explaining the diagnosis of a hematologic disease to minor children is correct and necessary. Opinions become radically different about the roles of professional figures: most Monza’s patients believe that a professional figure should be involved in communication, often in combination with parents, while most patients from other centers think that communication with children is an exclusive parents’ responsibility (*P* = .007). Moreover, the majority of other hospitals’ patients, where an intervention like Emanuela Project is not available, believe that the hematologist does not have a role in disease communication: this result is almost opposite to the Monza’s sample (*P* = .001).

**Table 6.** Monza versus other hospitals’ analysis—communication methods.

	Monza SP opinion, *n* (%)	Other centers SP opinion, *n* (%)	Chi-square test (*P*-value)
Is it correct to explain the disease to children?			
No	2 (5.9%)	2 (16.67%)	.375
Doesn’t know	0 (0%)	0 (0%)	
Yes	32 (94.1%)	10 (83.33%)	
Who should explain the disease to children?			
Parents	7 (21.9%)	7 (58.33%)	.007
Professional figure	6 (18.8%)	0 (0%)	
Both	19 (59.4%)	5 (41.67%)	
Can the hematologist have a role in communication?			
No	1 (2.9%)	6 (50%)	.001
Doesn’t know	1 (2.9%)	0 (0%)	
Yes	32 (94.1%)	6 (50%)	

Abbreviation: SP, sick parent.

## Discussion

The communication of a severe disease to patients’ minor children represents a major challenge.^18,20,21^

Data from the whole population showed that communicating a parent’s diagnosis of hematological disease to their minor children, even if different communication methods were used in the 4 centers, seems to have a positive impact, without any alarming change in their behavior at an early time point. An increase in attachment to both parents and some difficulties in separation from family figures were noticed.

Talking about the disease with minor children helps to create an open dialogue within the family about this difficult situation, and it renders unnecessary to keep hospital admissions and side effects hidden from children.^1,9^

At Monza, no significant differences were observed between sick and healthy parent’s opinion about children’s behavior, despite long periods of patient’s hospitalization: this result suggests that Emanuela Project, allowing frequent children’s visits to hospital and improving dialogue about the disease, helped patients to retain their parental role even in such a difficult period. We believe that familial and personal issues play a great role in the way patients deal with a life-threatening disease and should be properly addressed by clinicians.^1,24^

The comparison between Monza and other hospitals revealed some significant differences: a greater propensity to talk about the disease with family members was reported in Monza’s children, while a greater increase in attachment to healthy parent and more difficulties in separation from family figures were observed in the other centers’ group. These findings could be considered as positive effects of a well-established project on diagnosis communication to minor children, such as the Emanuela Project in Monza.

Almost all families believe in the importance of diagnosis communication to offspring, tearing down the old idea of protecting children from sufferance by keeping them in the dark.^13,16,17^ This finding is crucial, as it underlines the urgent need for hematologists to improve their knowledge about diagnosis communication with minors and to find the most appropriate way to address this patients’ necessity.^24^

Relevant differences were observed in the 2 groups regarding the professional figures involved in diagnosis communication, with a great majority of Monza’s patients supporting the involvement of professional figures, while other hospitals’ patients often considered that communication with children was mainly parents’ responsibility. Parents are strongly influenced by their own experience and by the opportunities they were offered or not. Parents from the other hospitals’ group often played the major role in diagnosis communication to children, and their opinions reflect their own experience, often not considering the role of the hematologist, instead crucial for Monza’s patients who took part in Emanuela Project.

We believe in the key role of the physician who takes care of the patient^1,24^: medical skills in communication are perceived by Monza’s patients as complementary to parents’ ones. Parents usually do not want to be replaced in their responsibilities,^15^ but they need to be supported by doctors in such a difficult task and they identify in their hematologist a trusted person who can take care of the whole family.

This new role of the hematologist in communication seems to have a positive impact also on patients, contributing to a better understanding of the disease and to increased trust in the medical staff, with a possible benefit in compliance to therapies during a long treatment course.

The study globally provided interesting results and offers the stimulus for further research, despite some limitations that should be mentioned:

(1) The small size of the study makes it hard to obtain statistically significant results.
(2) Questionnaires completed by parents are an important tool to explore children’s behavior, commonly used in this kind of studies, but answers given by parents are not objective measures. The effect of “self-report bias” on study results has been previously mentioned: parents’ opinion about diagnosis communication is influenced by their own experience and by opportunities they were offered or not.
(3) Another limitation of the study, especially concerning other hospitals’ group, regards lacking data from healthy parents, due to difficulties in questionnaires administration during COVID-19 pandemic.
(4) Long period of data collection can be considered also a limitation: a consistent part of questionnaires from Niguarda Hospital (3/7, 42.9%), Policlinico in Milano (3/4, 75%), and Policlinico San Matteo in Pavia (1/1, 100%) was administered during COVID-19 pandemic, which in Northern Italy has surely influenced hospital policies regarding children visits to Hematology wards. All questionnaires from San Gerardo Hospital in Monza were collected before COVID-19 pandemic.

Further research with larger cohorts of patients is needed, as well as the development of appropriate tools to obtain robust data in this peculiar field, including open questions and a nonquantitative statistical analysis.

The main take-home message from our investigation is that diagnosis communication to patient’s family, and especially to minor children, is a crucial moment in the process of taking care of patients with hematological neoplastic disease. The hematologist should not delegate this difficult task exclusively to parents or psychologists, as he/she can play a relevant role in diagnosis communication, with potential positive effects on patients’ compliance to treatment and on doctor-patient relationship.

## Supplementary material

Supplementary material is available at *The Oncologist* online.

oyae104_suppl_Supplementary_Materials

## Data Availability

The data underlying this article are available in the article and its online supplementary material.
