# Supplementary material for: Communicating the diagnosis of a hematological neoplastic disease to patients’ minor children: a multicenter prospective study
Source: Oncologist. 2024 May 22;29(10):e1354–63. doi: 10.1093/oncolo/oyae104 (PMC11449074; doi:10.1093/oncolo/oyae104)
Supplement: oyae104_suppl_Supplementary_Materials [file oyae104_suppl_supplementary_materials.zip › Supplementary materials/Supplemental Figures THE ONCOLOGIST.docx]

**SUPPLEMENTAL MATERIAL - FIGURES**

*
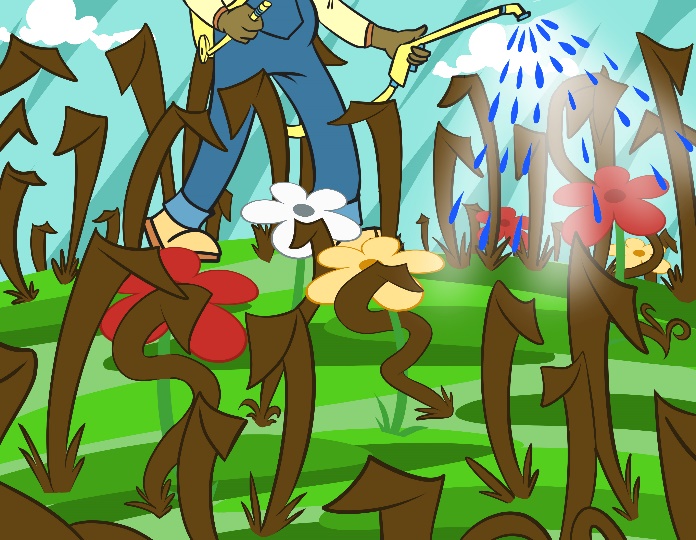
*

***Suppl. Figure 1:*** *Images used during diagnosis communication to patients’ children in Monza: the bone marrow is represented as a flowery meadow and leukemia blasts are weeds growing in the meadow.*

*
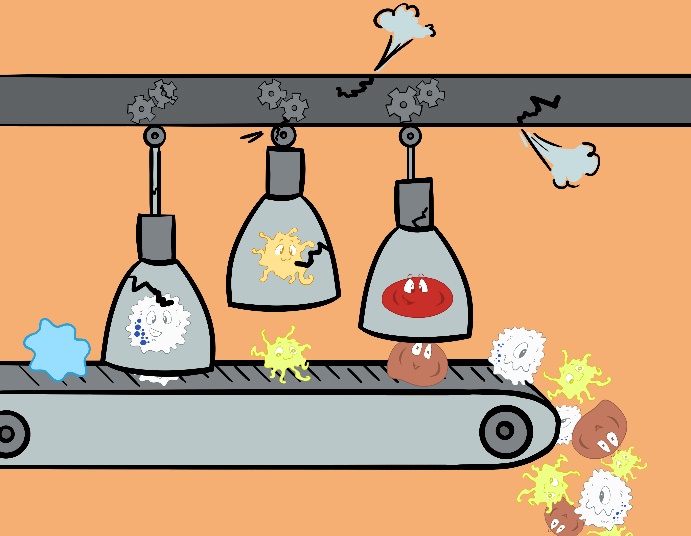
*

***Suppl. Figure 2:*** *Images used during diagnosis communication to patients’ children in Monza: the image of a factory with broken gears is used to explain myelodysplastic syndromes.*

*
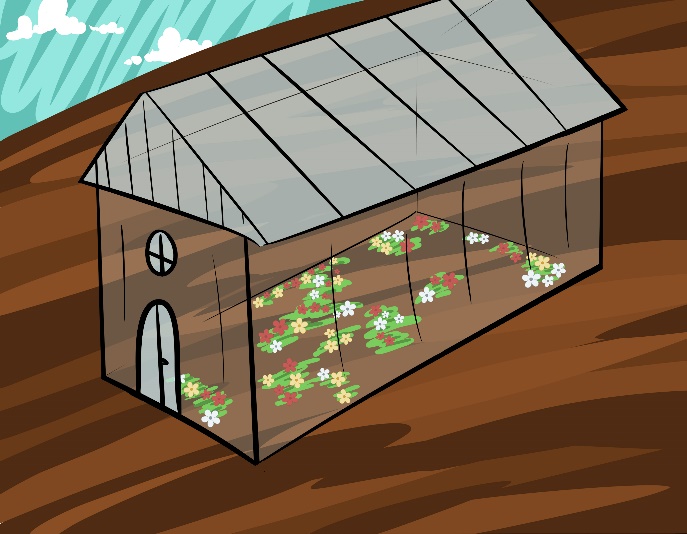
*

***Suppl. Figure 3:*** *Images used during diagnosis communication to patients’ children in Monza: the patient’s clean room is represented as a greenhouse which protects flowers from bad weather.*


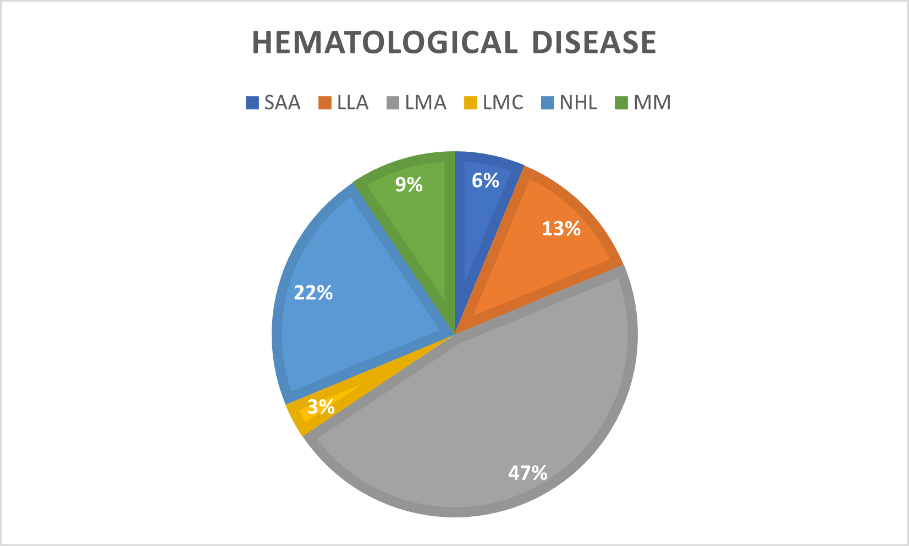


***Suppl. Figure 4:*** *Distribution of patients’ diagnoses in the study population.*

*SAA = Severe aplastic anemia*

*LLA = Acute lymphoblastic leukemia*

*LMA = Acute myeloid leukemia*

*LMC = Chronic myeloid leukemia*

*NHL = Non-Hodgkin lymphoma*

*MM = Multiple myeloma*


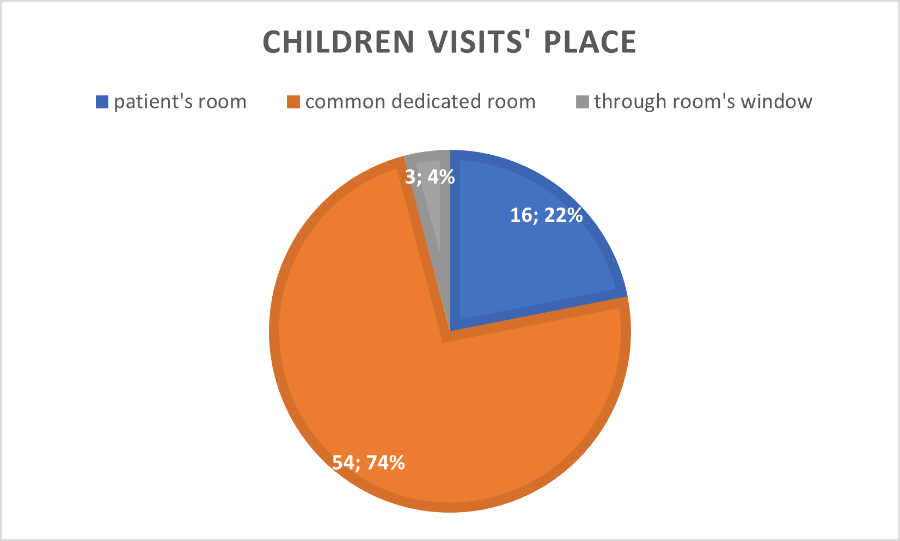


***Suppl. Figure 5:*** *Children visits’ place to hospitalized parents in the study population.*
