# Supplementary material for: Communicating the diagnosis of a hematological neoplastic disease to patients’ minor children: a multicenter prospective study
Source: Oncologist. 2024 May 22;29(10):e1354–63. doi: 10.1093/oncolo/oyae104 (PMC11449074; doi:10.1093/oncolo/oyae104)
Supplement: oyae104_suppl_Supplementary_Materials [file oyae104_suppl_supplementary_materials.zip › Supplementary materials/Supplemental material - Original questionnaires English.docx]

**SUPPLEMENTAL MATERIAL – ORIGINAL QUESTIONNAIRES (English)**

| ID CODE: |  |  |
| --- | --- | --- |

*Operator form*

**Information about the patient**

1) Disease:

2) Year of birth:

3) Nationality:

4) Date of diagnosis:

5) Has the patient undergone at least one hospitalization?

- Yes
- No

6) Duration of hospitalization:

7) Date of questionnaire administration:

**Information about patient’s children**

1) Number of children:

2) Children’s gender, year and month of birth:

3) Do the grandparents live together with the patient and his/her children?

**NOTES:** *Report any family situations (widowed parent, separated parent, foster or adopted children)*

**ID CODE INSTRUCTIONS**

- Coding of the operator form: code composed of the center abbreviation (e.g., MO for Monza) and the progressive number of the family in chronological order (01, 02, 03...).
- Coding of parents’ questionnaires: to the ID code assigned to the family is added:
- The letter A if it is completed by the father, uppercase if he is the sick parent
- The letter a if it is completed by the father, lower case if he is the healthy parent
- The letter B if it is completed by the mother, uppercase if she is the sick parent
- The letter b if it is completed by the mother, lower case if she is the healthy parent
- A sequential number (1,2,3...) to identify the child, starting with the eldest

| ID CODE |  |  |  |  |  |  |
| --- | --- | --- | --- | --- | --- | --- |

*Parents’ form*

**PART 1**

**A parent’s disease can cause deep changes in the parent-child relationship, triggering new behaviours and emotional states in children, and modifying the balance of the entire family unit.**

**We invite you to answer the following questions.**

**1)** During the hospitalization at the Hematology Department, was it possible for the children to visit the sick parent?

- Yes
- No
- The patient has not been hospitalized

**2)** If yes, how often? (no. of times per week)

**3)** If yes, where did the patient-children meetings take place?

**4)** Has your child been informed of the nature of mother/father's illness?

- Yes
- No

**4.1)** If yes, who was present at the time of the communication? *(It is possible to give more than one answer)*

| 1 | One or more siblings |
| --- | --- |
| 2 | Both parents |
| 3 | The healthy parent |
| 4 | The sick parent |
| 5 | A cultural mediator (translator) |
| 6 | Other professional figures |
| 7 | Other relatives or friends |
| 8 | Other people (please specify) …………………… |

**PART 2**

**After disease onset and communication, did you observe any changes in your child's behaviour and emotional state?**

1) Has there been any change in academic performance?

- Yes, it has improved
- Yes, it has worsened
- No, it is unchanged

2) Has there been any change in sleep pattern?

- Yes, he/she is sleeping more
- Yes, he/she is sleeping less
- No, it is unchanged

3) Has there been any change in appetite?

- Yes, it has increased
- Yes, it has decreased
- No, it is unchanged

4) Does he/she require the constant presence of the sick parent more often?

- Yes
- No

5) Does he/she require the constant presence of the healthy parent more often?

- Yes
- No

6) Does the child have more difficulty in separation from reference figures?

- Yes
- No

**In the following questions, you are asked to make a comparison, regarding some of your child's behaviours, between the period before and after the disease onset and communication.**

|  | Never | Sometimes | Often | Always |
| --- | --- | --- | --- | --- |
| 7a) In the past, he/she was used to play alone. | 1 | 2 | 3 | 4 |
| 7b) Do you think he/she now plays alone more often?   - Yes - No |  | | | |
| 8a) In the past, he used to wake up after having nightmares. | 1 | 2 | 3 | 4 |
| 8b) Do you think he/she now wakes up after having nightmares more often?   - Yes - No |  | | | |
| 9a) In the past, it happened that he/she talked about death. | 1 | 2 | 3 | 4 |
| 9b) Do you think he/she now talks about death more often?   - Yes - No |  | | | |

**The following questions refer to your child's attitude toward the parent's illness:**

|  | Never | Sometimes | Often | Always |
| --- | --- | --- | --- | --- |
| 10) He/she wants to be informed about the progress of the parent's illness | 1 | 2 | 3 | 4 |
| 11) He/she wants to talk about the parent’s illness | 1 | 2 | 3 | 4 |
| 12) He/she talks to others outside the family about mom/dad's illness. | 1 | 2 | 3 | 4 |
| 13) He/she talks to family members about mom/dad's illness | 1 | 2 | 3 | 4 |
| 14) In the family, it is possible to talk freely about the parent's illness | 1 | 2 | 3 | 4 |
| 15) Visits and hospitalizations of mom/dad are kept hidden in the family | 1 | 2 | 3 | 4 |
| 16) Any side effects of therapy are kept hidden in the family | 1 | 2 | 3 | 4 |

17) Does the child fear that something bad will happen to the sick parent?

- Yes
- No

18) Does the child fear that something bad will happen to other family members?

- Yes
- No

**PART 3**

**Please answer the following open-ended questions**

**1)** Can you think of a time when your child talked about the parent's illness in any way?

**2)** Have there been any changes in your child's behaviour since the onset of the illness? If so, what were they?

**3)** Can you describe a game, drawing or a small written thought of your child that you think has some relevance to the parent's illness? (You may wish to include a copy of the drawing)

**4)** Do you think it is right and useful to inform children about the diagnosis of their mother/father in language appropriate to their age?

**5)** Do you think this is the job of the parents themselves or of a specific professional figure?

**6)** Do you think the physician can play a role in this communication?

**7)** Do you have any suggestions?

| ID CODE: |  |  |  |  |  |  |
| --- | --- | --- | --- | --- | --- | --- |

**PART 4**

**Below you will find some open questions related to the interview conducted by the hematologist and psychologist with your child.**

**1)** Did the doctor seem to explain the illness and treatment in a simple and clear way?

**2)** How do you rate the tools used to promote dialogue with children?

**3)** Did you find the hematologist's role in the interview useful?

**4)** Did you find the psychologist’s role in the interview useful?

**5)** Do you think that this interview, carried out by the hematologist and the psychologist, was helpful for the child overall?

**6)** After the interview, do you think it is right and useful to tell the children about the mother/father's diagnosis in age-appropriate language?

**7)** Did the pictures and examples during the interview help you to understand the disease and treatment?

**8)** Do you have the feeling that your relationship with health care professionals (doctors, nurses) has improved as a result of this experience?
