# Supplementary material for: Communicating the diagnosis of a hematological neoplastic disease to patients’ minor children: a multicenter prospective study
Source: Oncologist. 2024 May 22;29(10):e1354–63. doi: 10.1093/oncolo/oyae104 (PMC11449074; doi:10.1093/oncolo/oyae104)
Supplement: oyae104_suppl_Supplementary_Materials [file oyae104_suppl_supplementary_materials.zip › Supplementary materials/Supplemental Tables THE ONCOLOGIST.docx]

**SUPPLEMENTAL MATERIAL – TABLES**

***Suppl. Table 1: Monza patients’ analysis – Communication methods***

|  | **Sick parent’s opinion,**  **n (%)** | **Healthy parent’s opinion,**  **n (%)** | **Mc Nemar test** |
| --- | --- | --- | --- |
| **Is it correct to explain the disease to children?**   - No - Doesn’t know - Yes | 2 (5.9%)  0 (0%)  32 (94.1%) | 0 (0%)  0 (0%)  32 (100%) | p = NA |
| **Who should explain the disease to children?**   - Parents - Professional figure - Both | 7 (21.9%)  6 (18.8%)  19 (59.4%) | 5 (16.1%)  5 (16.1%)  21 (67.7%) | p = 1.00 |
| **Can the hematologist have a role in communication?**   - No - Doesn’t know - Yes | 1 (2.9%)  1 (2.9%)  32 (94.1%) | 1 (3.1%)  0 (0%)  31 (96.9%) | p = 1.00 |

***Suppl. Table 2: Monza vs other hospitals’ analysis – Family dialogue about the disease***

|  | **Monza SP* opinion, n (%)** | **Other centres SP* opinion, n (%)** | **Chi square test** |
| --- | --- | --- | --- |
| **Children want information about course of disease:**   - Never - Sometimes - Often - Always | 4 (11.76%)  18 (52.94%)  8 (23.53%)  4 (11.76%) | 1 (8.33%)  7 (58.33%)  3 (25%)  1 (8.33%) | p = 0.895 |
| **Children want to talk about the disease:**   - Never - Sometimes - Often - Always | 10 (29.41%)  19 (55.88%)  3 (8.82%)  2 (5.88%) | 2 (16.67%)  8 (66.67%)  1 (8.33%)  1 (8.33%) | p = 0.497 |
| **Children talk about disease outside family:**   - Never - Sometimes - Often - Always | 7 (21.88%)  20 (62.5%)  4 (12.5%)  1 (3.13%) | 7 (58.33%)  5 (41.67%)  0 (0%)  0 (0%) | p = 0.256 |
| **Children talk about disease with family members:**   - Never - Sometimes - Often - Always | 8 (25%)  15 (46.88%)  9 (28.13%)  0 (0%) | 5 (41.67%)  7 (58.33%)  0 (0%)  0 (0%) | p = 0.067 |
| **Free dialogue about the disease in the family:**   - Never - Sometimes - Often - Always | 0 (0%)  6 (17.65%)  7 (20.59%)  21 (61.76%) | 1 (8.33%)  3 (25%)  3 (25%)  5 (41.67%) | p = 0.304 |
| **Need to hide parent’s visits and hospitalization:**   - Never - Sometimes - Often - Always | 34 (100%)  0 (0%)  0 (0%)  0 (0%) | 11 (91.67%)  1 (8.33%)  0 (0%)  0 (0%) | p = NA |
| **Need to hide side effects of therapies:**   - Never - Sometimes - Often - Always | 23 (67.65%)  8 (23.35%)  2 (5.88%)  1 (2.94%) | 9 (75%)  3 (25%)  0 (0%)  0 (0%) | p = 0.272 |
| **Children fear for sick parent’s life:**   - No - Yes | 16 (47.06%)  18 (52.94%) | 8 (66.67%)  4 (33.33%) | p = 0.176 |
| **Children fear for healthy parent’s life:**   - No - Yes | 24 (75%)  8 (25%) | 11 (91.67%)  1 (8.33%) | p = 0.191 |

*SP = sick parent
